# Differential Regulation of Hepatic Transcription Factors in the Wistar Rat Offspring Born to Dams Fed Folic Acid, Vitamin B_12_ Deficient Diets and Supplemented with Omega-3 Fatty Acids

**DOI:** 10.1371/journal.pone.0090209

**Published:** 2014-02-28

**Authors:** Akshaya Meher, Asmita Joshi, Sadhana Joshi

**Affiliations:** Department of Nutritional Medicine, Interactive Research School for Health Affairs, Bharati Vidyapeeth University, Pune, Maharashtra, India; CIMA. University of Navarra, Spain

## Abstract

Nutritional status of the mother is known to influence various metabolic adaptations required for optimal fetal development. These may be mediated by transcription factors like peroxisome proliferator activated receptors (PPARs), which are activated by long chain polyunsaturated fatty acids. The objective of the current study was to examine the expression of different hepatic transcription factors and the levels of global methylation in the liver of the offspring born to dams fed micronutrient deficient (folic acid and vitamin B_12_) diets and supplemented with omega-3 fatty acids. Female rats were divided into five groups (n = 8/group) as follows; control, folic acid deficient (FD), vitamin B_12_ deficient (BD) and omega-3 fatty acid supplemented groups (FDO and BDO). Diets were given starting from pre-conception and continued throughout pregnancy and lactation. Pups were dissected at the end of lactation. Liver tissues were removed; snap frozen and stored at −80°C. Maternal micronutrients deficiency resulted in lower (p<0.05) levels of pup liver docosahexaenoic acid (DHA) and arachidonic acid (ARA) as compared to the control group. Pup liver PPARα and PPARγ expression was lower (p<0.05) in the BD group although there were no differences in the expression of SREBP-1c, LXRα and RXRα expression. Omega-3 fatty acids supplementation to this group normalized (p<0.05) levels of both PPARα and PPARγ but reduced (p<0.05) SREBP-1c, LXRα and RXRα expression. There was no change in any of the transcription factors in the pup liver in the FD group. Omega-3 fatty acids supplementation to this group reduced (p<0.05) PPARα, SREBP-1c and RXRα expression. Pup liver global methylation levels were higher (p<0.01) in both the micronutrients deficient groups and could be normalized (p<0.05) by omega-3 fatty acid supplementation. Our novel findings suggest a role for omega-3 fatty acids in the one carbon cycle in influencing the hepatic expression of transcription factors in the offspring.

## Introduction

Nutritional imbalance during pregnancy influences metabolic programming of the fetus and is likely that transcription factors like peroxisome proliferator activated receptors (PPARs) play a key role by regulating expression of genes that are important to lipid homeostasis and metabolism [1]–[4]. Perturbations in the expression of these factors in the liver are associated with cardiovascular and metabolic disturbances [5]. PPARs are ligand activated nuclear transcription factors and regulate the expression of numerous genes involved in fatty acid, carbohydrate and cholesterol metabolism [6], [7]. The other transcription factors regulating the fatty acid metabolism are liver X receptor α (LXRα), retinoid X receptor (RXR) and the sterol regulatory element binding protein (SREBP-1c) [8]–[11].

Maternal high multivitamin intakes (10 fold increase in the AIN-93G diet) [12], 50% caloric restriction [13], protein restricted diet [14], n-6 and n-3 polyunsaturated fatty acids (PUFAs) [15], [16] and folic acid supplementation [17], during pregnancy and/or lactation period have been reported to alter the levels of PPAR in different fetal tissues like brain, placenta, liver and lung.

Long chain polyunsaturated fatty acids (LCPUFAs) like docosahexaenoic acid (DHA) are known to activate many of these transcription factors [18]. These fatty acids regulate hepatic energy metabolism by either up or down regulating the expression of a whole set of genes involved in fatty acid synthesis (LXRα and SREBP-1) and fatty acid oxidation (PPARα) [19]. A recent review indicates that eicosapentaenoic acid (EPA) and DHA alter the membrane fluidity and thereby interacts with PPAR and SREBP and improves cardiovascular health by altering lipid metabolism [20]. However, there are no studies which have examined the effect of maternal micronutrient deficiencies during pre-conception on hepatic nuclear transcription factors in the offspring.

Our earlier studies in animals have shown that an imbalance in maternal micronutrients during pregnancy leads to reduced LCPUFA levels and altered global methylation in the placenta [21]. We have extensively demonstrated that folic acid; vitamin B_12_ and omega-3 fatty acids are interlinked in the one carbon cycle and the adverse effects of a maternal micronutrient deficient/imbalanced diet on the fatty acid metabolism in the liver and placenta could be ameliorated by the supplementation of omega-3 fatty acids during pregnancy [22], [23]. Taking these studies further we hypothesize that the expression of transcription factors in the liver of the offspring will be determined by the maternal micronutrients through one carbon cycle and the omega-3 fatty acid status.

Recent studies indicate that maternal pre-conception nutritional status of a woman is an important determinant of fetal growth and development [24], [25]. The objective of the current study was to examine the expression of different hepatic transcription factors and the levels of global methylation in the liver of the offspring born to dams fed micronutrient deficient (folic acid and vitamin B_12_) diets and supplemented with omega-3 fatty acids.

## Materials and Methods

The current study was carried out in strict accordance with the CPCSEA guidelines (Committee for the purpose of control and supervision of experimental animals) Government of India and was approved by the Bharati Vidyapeeth Animal Ethical Committee (2/2011/CPCSEA). All efforts were made to minimize suffering.

### i. Animals

Female Wistar rats (n = 8 per group) weighing 45 g were randomly divided into 5 different groups starting from pre-pregnancy and were put for breeding after gaining 200 g of weight (approximately 7 weeks). Diets were continued throughout pregnancy and lactation. The composition of the control and the treatment diets was as per AIN 93-G purified diets for laboratory rodents [26] and has been shown in the table 1 and have been reported by us earlier [27]. Briefly, there were 4 treatment diets, folic acid deficient (FD), vitamin B_12_ deficient (BD), folic acid deficient + omega-3 fatty acid supplemented (FDO) and vitamin B_12_ deficient + omega-3 fatty acid supplemented (BDO). In the treatment groups, the diet was formulated as per the AIN 93-G guidelines; however in the vitamin mixture the folic acid or vitamin B_12_ was completely omitted. Also, vitamin free casein was used for all the treatment diets. Further, rats receiving vitamin B_12_ and folic acid deficient diets were kept in special cages to prevent coprophagy. Thus, folic acid deficiency and vitamin B_12_ deficiency was obtained exclusively through dietary means. The dietary deficiencies resulted in significantly lower levels of micronutrients in the dam plasma and have been reported by us earlier [27].

**Table 1:** Composition of the Diets.

Diets	Control	BD	FD	BDO	FDO
(g/kg)					
Corn Starch	398	398	398	398	398
Casein (>85% protein)	200	200	200	200	200
Dextrinized Starch	132	132	132	132	132
Sucrose	100	100	100	100	100
Soya Bean Oil	70	70	70	25	25
Omega-3 fatty acids	0	0	0	45	45
Fiber	50	50	50	50	50
Mineral mixture^a^	35	35	35	35	35
Vitamin mixture^b^	10	10	10	10	10
Folic acid (mg)	2	2	0	2	0
Vitamin B_12_ (in 0.1% Mannitol)	2.5	0	2.5	0	2.5
Cystine	3	3	3	3	3
Choline Bitartarate	2.5	2.5	2.5	2.5	2.5
Tertiary Butyl Hydroquinone	0.014	0.014	0.014	0.014	0.014
Total Energy (kcal)	3766.0	3766.0	3766.0	3766.0	3766.0

a Mineral mixture (g/kg mixture):Calcium carbonate, 357; Potassium Phosphate, 196; Potassium Citrate, 70.78; Sodium Chloride, 78; Potassium Sulphate, 46.6; Magnesium Oxide, 24; Ferric Citrate, 6.06; Zinc Carbonate, 1.65; Manganous Carbonate, 0.63; Cupric Carbonate, 0.3; Potassium Iodate, 0.01; Sodium Selenate, 0.01; Ammonium Paramolybdate, 0.007; Sodium Metasilicate, 1.45; Chromium Potassium Sulphate, 0.275; Lithium Chloride, 0.01; Boric Acid, 0.08; Sodium Fluoride, 0.06; Nickel Carbonate, 0.03; Ammonium Vanadate, 0.006; Sucrose, 221.02.

b Vitamin mixture (g/kg mixture):Nicotinic Acid, 3; Calcium Pantothenate, 1.6; Pyridoxine-HCl, 0.7; Thiamin HCl, 0.6; Riboflavin, 0.6; D-Biotin, 0.02; Vitamin B_12_ (in 0.1% Mannitol), 2.5;Vitamin E, 15;Vitamin A, 0.8; Vitamin D-3, 0.25;Vitamin K, 0.075; Folic acid, 0.2 (control) and Sucrose 974.655, was used to make total weight of the vitamin mixture to 1 kg.

Control- Normal folic acid, normal vitamin B_12_; BD- vitamin B_12_ deficient; FD- folic acid deficient; BDO- vitamin B_12_ deficient and omega-3 fatty acid supplemented; FDO- folic acid deficient and omega-3 fatty acid supplemented.

Maxepa (fish oil, MERCK LIMITED, Goa, India. Catalog no- 614400612) which contained a combination of DHA (120 mg) and EPA (180 mg) was used as the source of omega-3 fatty acids. The maxepa capsules were added in the diet composition and given as a feed. 45 mL of maxepa was added per kg of diet. Animals consumed approximately 30 g of diet per day. The time taken for breeding was different for females in different groups. In general, the female rats in control, BDO and FDO group mated within 1 week, however those in BD and FD group, took longer time (approximately 10 days) and has been reported by us earlier [27]. The dams were allowed to deliver by spontaneous vaginal delivery and the litter size was culled to eight to maintain nutritional adequacy. All the pups were dissected on the postnatal day 22. Pups were anesthetized with anesthetic ether and blood was drawn from the heart of the pups using the syringe (2 mL) and collected in the tubes containing ethylene diamine tetraacetic acid (EDTA). Plasma was separated and stored at −80°C. Plasma samples were used for the folic acid and vitamin B_12_ estimations. Liver tissues were removed and snap frozen and stored at −80°C until further use. Daily feed intake of dams and weekly weights of all the pups were recorded during lactation.

### ii. Liver weights

The absolute weight of the liver was recorded on a Schimadzu electronic balance with a least count of 0.001 g. These liver samples were immediately snap frozen in liquid nitrogen and stored at −80°C for biochemical estimations. The relative liver weights were expressed as [(absolute liver weight/weight of the pup)* 100].

### iii. Folic acid and vitamin B_12_ estimations

Pup plasma folic acid and vitamin B_12_ concentrations were determined using Chemiluminiscent Microparticle Immunoassay Technology (Abbott Diagnostics, Lisnamuck, Longford, Co. Longford, Ireland). The reference range used in the assay was 2.34–17.56 ng/mL for folic acid and 187–883 pg/mL for vitamin B_12_.

### iv. Liver Fatty acids levels

Fatty acids were estimated from the pup liver samples using the gas chromatography. The method has been described by our lab in previous studies [21]–[23]. Fatty acids were expressed as g/100 g total fatty acid.

### v. Pup liver global DNA Methylation

Genomic DNA extraction from pup liver tissues was carried out with the Qiagen Blood and Tissue kit (Qiagen, Hilden, Germany, Catalog no- 69504). Global DNA methylation was measured using the MethylampTM Global DNA Methylation Quantification Kit (Epigentek Group Inc., New York, NY, U.S.A, Catalog no. P-1034) as described by us earlier [21]. The kit yields accurate measures of methyl cytosine content as a percentage of total cytosine content. The methodology for estimation of global methylation levels used in this study takes into account methylation of all CpG's irrespective of their position in the genome (promoter and non promoter CpG).

### vi. RNA isolation, cDNA synthesis and pup liver gene expression

Total RNA was isolated from liver tissue using Trizol reagent (Invitrogen, Van Allen Way, Carlsbad CA, USA. Catalog no- 15596026) and was quantified by using Biophotometer (Eppendorf AG, Hamburg, Germany, Catalog no-22331). Reverse transcription was carried out with oligo(dT) primer and Super Script II reverse transcriptase from 1 µg of total RNA using High-Capacity cDNA reverse transcription Kit (Applied Biosystems, Foster City, CA, USA, Catalog no- 4368814).

Real-time quantitative PCR for the PPAR*γ*, PPARα, SREBP-1c, LXRα, and RXRα genes was performed using the Applied Biosystems 7500 Standard system. The relative expression level of the gene of interest was computed with respect to Glyceraldehyde-3-phosphate dehydrogenase (GAPDH) mRNA to normalize for variation in the quality of RNA and the amount of input cDNA. Real-time PCR was performed with the TaqMan Universal PCR Master Mix (Applied Biosystems, Brachburg, New Jersey, USA. Catalog no- 4324018) using cDNA equivalent to 100 ng total RNA. ΔCT values corresponded to the difference between the CT-values of the GAPDH (internal control) gene and those of respective genes. Relative expression levels of genes were calculated and expressed as 2^ΔCT^ The following TaqMan® assays (Applied Biosystems, Foster City, CA, USA) were used in this study: GAPDH (Rn99999916_S1), PPARγ (Rn00440945_M1), PPARα (Rn00566193_M1), SREBP-1c (Rn01495769_M1), LXRα (Rn00581185_M1) and RXRα (Rn00441185_M1).

### vii.Statistical Analysis

Litter means were used as the unit of analysis. Results are presented as mean ± SD (standard deviation). The data was analyzed using SPSS/PC+ package (Version 20.0, Chicago IL). The treatment groups were compared with the control group by ANOVA and the post-hoc least significant difference test. Data was normalized by using log transformation.

## Results

### i. Intake

The weekly food intakes (g) of the dams during lactation are described in table 2. The average intake of dams during lactation was significantly lower (p<0.05 for both) in the FDO group as compared to control as well as compared to FD group. The average intake in FD, BD and BDO group was comparable with the control group.

**Table 2:** Intakes (g) of animals in different groups during lactation period.

Group	Lact week 1	Lact week 2	Lact week 3	Avg lact intake
	(n = 8)	(n = 8)	(n = 8)	(n = 8)
**Control**	22.08±3.09	32.12±7.09	45.87±12.17	33.35±6.68
**FD**	21.40±4.66	34.67±3.85	44.01±7.38	33.36±4.33
**FDO**	16.53±4.67 ^**$$^	29.26±4.18	35.92±6.43	27.24±3.84 * **^$^**
**BD**	21.46±4.43	28.29±8.39	36.72±13.58	28.83±7.83
**BDO**	20.44±2.04	32.34±6.83	41.22±10.05	31.33±5.82

The table shows the intake of dams in the different groups. The treatment groups were compared with the control group by One Way ANOVA and the post-hoc least significant difference test. Results are presented as mean ± SD (standard deviation).

^*^p<0.05 as compared to control;^**^p<0.01 as compared to control; **^$^** p<0.05 as compared to FD group; **^$$^** p<0.01 as compared to FD group.

Control – Normal folic acid, normal vitamin B_12_; FD- Folic acid deficient; BD- Vitamin B_12_ deficient; FDO- folic acid deficient and omega-3 fatty acid supplemented; BDO- vitamin B_12_ deficient and omega-3 fatty acid supplemented.

### ii. Average pup weights

Average pup weights at birth in all the treatment groups were lower (p<0.05 for all) than control. At day 21 of lactation, the average pup weight in the BD (p<0.01), FDO (p<0.01) as well as BDO group (p<0.01) was lower as compared to control group. Pup weight in the FDO group was lower (p<0.01) as compared to the FD group while it was higher (p<0.01) in the BDO group as compared to the BD group (Table 3).

**Table 3:** Average pup weight (g) in different groups during lactation.

Group	At birth	Day 7	Day 14	Day 21
**Control**	7.9±1.1	16.6±1.8	32.3±4.4	48.8±5.1
**FD**	6.7±0.4 ^**^	15.1±1.6	32.1±4.2	46.2±7.5
**FDO**	6.9±0.5 *	14.8±2.0	28.9±3.9	41.6±7.0^**$$^
**BD**	6.3±0.8 ^**^	13.4±2.0 ^**^	25.7±4.1 ^**^	36.8±6.9^**^
**BDO**	6.5±0.5 ^**^	15.0±1.7	27.9±4.4 *	39.7±8.1 ^**@^

Data in each group is on 4 males and 4 females. The treatment groups were compared with the control group by One Way ANOVA and the post-hoc least significant difference test. Results are presented as mean ± SD (standard deviation).

^*^p<0.05 as compared to control; ^**^p<0.01 as compared to control; **^@^** p<0.05 as compared to BD group; **^$$^** p<0.01 as compared to FD group.

Control – Normal folic acid, normal vitamin B_12_; FD- Folic acid deficient; BD- Vitamin B_12_ deficient; FDO- folic acid deficient and omega-3 fatty acid supplemented; BDO- vitamin B_12_ deficient and omega-3 fatty acid supplemented.

### iii. Absolute and Relative Liver weights

The absolute liver weights of animals in BD group (1.24±0.32 g) was lower (p<0.01 for both) as compared to control group (1.84±0.26 g) as well as BDO group (1.50±0.37 g). Also, the absolute liver weights of animals in FD group (1.61±0.32 g), FDO group (1.65±0.33 g) and BDO group were lower (p<0.01 for all) as compared to control group.

The relative liver weight of animals in both the micronutrient deficient groups i.e FD (3.46±0.31) and BD (3.36±0.51) were lower (p<0.01 for both) as compared to control group (3.79±0.51). Further omega-3 fatty acid supplementation i.e both FDO (3.98±0.63) and BDO (3.77±0.52) group increased (p<0.01 for both) the relative liver weight of animals as compared to their respective micronutrient deficient groups.

### iv. Folic acid and vitamin B_12_ levels in pup plasma

Pup plasma folic acid levels were lower (p<0.01) in the FD group (10.13±3.30 ng/mL) as compared to control group (43.94±19.94 ng/mL). Further, these levels were lower (p<0.01) in the FDO group (4.86±2.28 ng/mL) as compared to the control group. The plasma folic acid levels in the BD group (18.20±4.36 ng/mL) and BDO group (19.46±1.07 ng/mL) were also lower (p<0.01) as compared to the control group. Plasma folic acid levels were different between FD and FDO groups which may possibly be due to the fact that folic acid and omega-3 fatty acids are interlinked in the one carbon cycle. Further studies are needed to understand mechanisms.

Pup plasma vitamin B_12_ levels were lower (p<0.01 for all) in all the treatment groups i.e. FD (504.75±96.02 pg/mL), BD (104.25±28.72 pg/mL), FDO (622.00±103.87 pg/mL) and BDO (91.88±13.46 pg/mL) as compared to the control group (846.63±208.10 pg/mL).

### v. Pup liver fatty acids

The levels of 15 different fatty acids in different groups are as described in table 4. The levels of DHA were lower (p<0.05) in the both FD and BD group as compared to the control. Omega-3 fatty acid supplementation to the micronutrient deficient groups i.e FDO and BDO increased (p<0.01 for all) the levels of DHA as compared to their respective deficiency group as well as compared to control.

**Table 4:** Pup liver fatty acid levels (g/100 g total fatty acids) in different treatment groups.

	Control	FD	BD	FDO	BDO
			Mean ± SD		
MYR	1.15±0.53	1.84±0.44 ^**^	1.40±0.52	1.34±0.52 ^$^	1.14±0.26
MYRO	0.04±0.01	0.08±0.06	0.07±0.06	0.03±0.02^$^	0.04±0.03
PAL	18.42±0.98	17.38±1.16	17.45±0.95	21.88±1.42^**$$^	20.94±2.26^**@@^
PALO	0.52±0.14	0.62±0.26	0.62±0.28	0.59±0.12	0.66±0.12
STE	17.49±1.44	14.70±2.22^**^	14.94±2.24*	15.46±2.58*	14.74±0.68^**^
OLE	7.80±1.04	9.36±1.83*	8.70±2.26	5.20±1.10 ^**$$^	5.62±0.77^**@@^
LA	18.67±1.23	22.94±2.53^**^	21.11±2.54*	10.01±0.65 ^**$$^	10.04±1.55 ^**@@^
ALA	0.63±0.13	0.90±0.29^**^	0.74±0.20	0.25±0.11 ^**$$^	0.28±0.10 ^**@@^
GLA	0.21±0.12	0.38±0.26*	0.21±0.10	0.06±0.04 ^**$$^	0.06±0.03 ^**@@^
DGLA	0.46±0.08	0.57±0.13	0.74±0.25 ^**^	0.74±0.14 ^**$^	0.74±0.11 ^**^
ARA	21.39±3.66	17.86±1.71 ^**^	18.42±1.10 ^**^	7.66±1.82 ^**$$^	7.74±0.95 ^**@@^
EPA	0.30±0.14	0.39±0.16	0.96±1.34	5.37±0.72 ^**$$^	5.57±1.13 ^**@@^
NA	1.47±0.32	1.03±0.17 ^**^	1.45±1.35	0.16±0.04 ^**$$^	0.13±0.05 ^**@@^
DPA	1.17±0.30	1.06±0.29	1.26±0.30	5.12±1.64 ^**$$^	4.60±1.04 ^**@@^
DHA	6.21±.95	5.41±0.72 *	5.47±0.88 *	22.37±1.55 ^**$$^	21.69±1.34 ^**@@^
Omega-3 fatty acids	7.14±0.79	6.71±0.48	7.16±1.56	28.00±1.81 ^**$$^	27.54±2.29^**@@^
Omega-6 fatty acids	41.91±3.87	42.80±1.62	41.74±2.44	23.58±1.40 ^**$$^	23.17±1.59^**@@^
MUFA	9.82±1.10	11.08±2.06	10.83±1.83	5.98±1.25 ^**$$^	6.44±0.81 **@@
SFA	37.06±1.76	33.91±2.52 ^**^	33.79±1.95 ^**^	38.68±1.99 ^$$^	36.83±2.43 ^@@^

Data in each group is on 4 males and 4 females. The treatment groups were compared with the control group by One Way ANOVA and the post-hoc least significant difference test. Results are presented as mean ± SD (standard deviation).

^*^p<0.05 as compared to control; ^**^p<0.01 as compared to control; ^$^p<0.05 as compared to FD group; ^$$^p<0.01 as compared to FD group; ^@^p<0.05 as compared to BD group; ^@@^p<0.01 as compared to BD group

MYR- Myristic acid, MYRO- Myristoleic acid, PAL- Palmitic acid, PALO- Palmitoleic acid, STE- Stearic acid, OLE- Oleic acid, LA- Linoleic acid, GLA- γ Linolenic acid, ALA- α Linolenic acid, DGLA- di homo γ linolenic acid, ARA- Arachidonic acid, EPA-Eicosapentaenoic acid, NA- Nervonic acid, DPA- Docosapentaenoic acid, DHA-Docosahexaenoic acid, MUFA- Mono unsaturated fatty acids, SFA-Saturated fatty acids.

Control – Normal folic acid, normal vitamin B_12_; FD- Folic acid deficient; BD- Vitamin B_12_ deficient; FDO- folic acid deficient and omega-3 fatty acid supplemented; BDO- vitamin B_12_ deficient and omega-3 fatty acid supplemented.

The levels of arachidonic acid (ARA) were lower in both the micronutrient deficient groups i.e. FD (p<0.01) and BD (p<0.05) as compared to control group. Omega-3 fatty acid supplementation decreased the levels of ARA in FDO group as compared to FD group (p<0.01) as well as compared to control group (p<0.01) and in BDO group as compared to BD group (p<0.01) as well as compared to control group (p<0.01).

### vi. Global DNA Methylation

**Figure 1:**
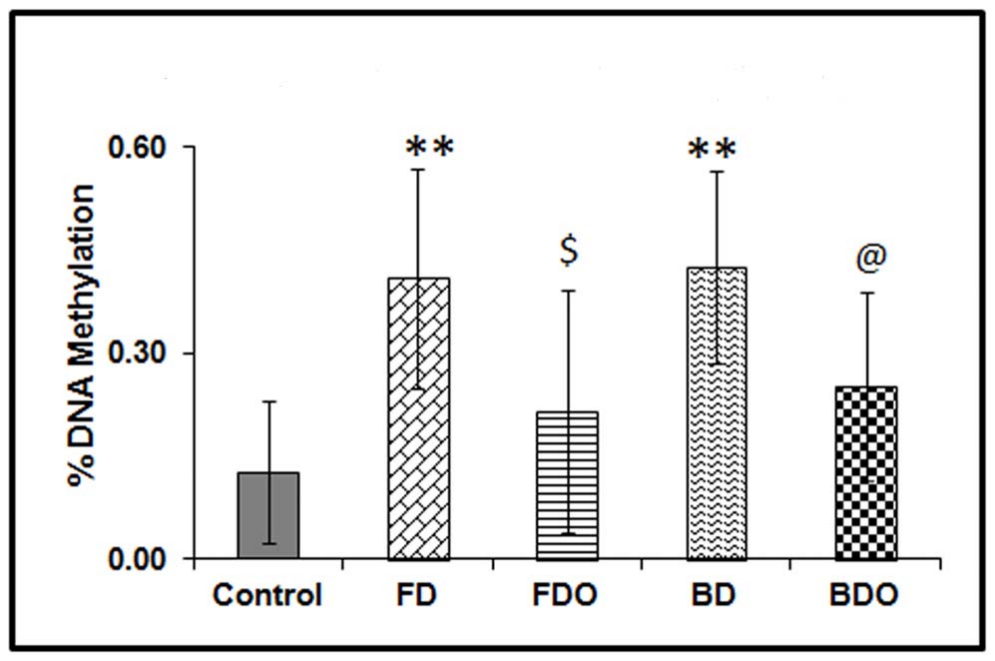
Pup liver global DNA methylation in different groups. Data in each group is on 4 males and 4 females. The treatment groups were compared with the control group by One Way ANOVA and the post-hoc least significant difference test. Results are presented as mean ± SD (standard deviation). **p<0.01 as compared to control; $p<0.05 as compared to FD group; @ p<0.05 as compared to BD group. Control – Normal folic acid, normal vitamin B_12_; FD– folic acid deficient; BD – vitamin B_12_ deficient; FDO– folic acid deficient and omega-3 fatty acid supplementation; BDO– vitamin B_12_ deficient and omega-3 fatty acid supplementation.

In case of pup liver global DNA methylation, the levels were higher (p<0.01) in both the micronutrient deficient groups (FD and BD) as compared to control group. Omega-3 fatty acid supplementation to these groups (FDO and BDO) reduced (p<0.05) the levels of global DNA methylation as compared to their respective micronutrient deficient groups (Fig. 1).

### vii. Pup liver gene expression

**Figure 2:**
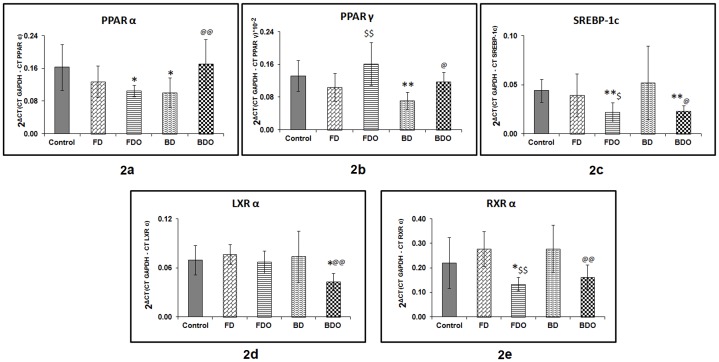
Pup liver gene expression in different groups. Figure 2a- Pup liver PPARα expression in different groups. Figure 2b- Pup liver PPARγ expression in different groups. Figure 2c- Pup liver SREBP-1c expression in different groups. Figure 2d- Pup liver LXRα expression in different groups. Figure 2e- Pup liver RXRα expression in different groups. Gene expression was carried out in pup liver using real time PCR. Data in each group is on 4 males and 4 females. The treatment groups were compared with the control group by ANOVA and the post-hoc least significant difference test. Data is presented as the mean ± SD of 2^ΔCT^ where ΔCT is CT (GAPDH) – CT (target gene). *p<0.05 as compared to control; **p<0.01 as compared to control; $p<0.05 as compared to FD group; $$p<0.01 as compared to FD group; @ p<0.05 as compared to BD group; @@ p<0.01 as compared to BD group. Control – Normal folic acid, normal vitamin B_12_; FD– folic acid deficient; BD – vitamin B_12_ deficient; FDO– folic acid deficient and omega-3 fatty acid supplementation; BDO– vitamin B_12_ deficient and omega-3 fatty acid supplementation.

Pup liver PPARα expression was lower in the BD (p<0.05) and FDO groups (p<0.05) as compared to control group. Omega-3 fatty acid supplementation to the vitamin B_12_ deficient diet (BDO group) increased (p<0.01) the expression of PPARα in the pup liver tissue as compared to BD group. However, in case of FDO group, there was no difference in the expression of PPARα as compared to FD group (Fig. 2a).

Pup liver PPARγ expression was lower (p<0.01) in the BD group as compared to control group. Omega-3 fatty acid supplementation to the micronutrient deficient diet [FDO (p<0.01) and BDO group (p<0.05)] increased the expression of PPARγ as compared to their respective deficiency groups (Fig. 2b).

There was no difference observed in the pup liver SREBP-1c expression between the micronutrient deficiency groups (FD and BD) and the control group. However, omega-3 fatty acid supplementation to the micronutrient deficient diet (FDO and BDO group) decreased the expression of SREBP-1c as compared to their respective deficiency groups (p<0.05 for both) as well as compared to control group (p<0.01 for both) (Fig. 2c).

Pup liver LXRα and RXRα expression was similar in both the micronutrient deficient groups (FD and BD), as compared to the control group. Omega-3 fatty acid supplementation decreased the expression of LXRα significantly in BDO group as compared to control (p<0.05) as well as compared to BD group (p<0.01). No difference was observed in case of FDO group as compared to FD group (Fig. 2d). In case of RXRα, omega-3 fatty acid supplementation to the micronutrient deficient diet (FDO and BDO group) decreased (p<0.01) the expression of RXRα as compared to their respective deficiency group as well as compared to control (p<0.05) in case of FDO group (Fig. 2e).

## Discussion

To our knowledge this is the first report which has examined the effect of folic acid and vitamin B_12_ deficiency from pre-conception on the expression of various transcription factors in the pup liver. The study also examined the effect of omega-3 fatty acid supplementation to these micronutrient deficient diets. Our results indicate that maternal micronutrient deficiency (folic acid and vitamin B12) resulted in the following changes in the pup liver 1) lower expression of PPARα, PPARγ genes 2) No change in the expression of SREBP-1c, LXRα, RXRα expression in the pup liver and 3) Increased levels of pup liver global DNA methylation. Omega-3 fatty acid supplementation was able to normalize most of the effects observed as a result of vitamin B12 and folic acid deficiency.

In the current study, the expression of PPARα and PPARγ genes in the pup liver was significantly lower as a consequence of maternal micronutrient deficiency. Studies have reported that dietary manipulation in pregnant rats alters the hepatic PPARα gene expression in the offspring [5], [14], [28]–[29]. It is well known that dietary PUFAs regulate the abundance of these nuclear transcription factors [30]. Thus, it may be possible that the decrease in the levels of DHA and ARA may be associated with the lower expression of PPARs in the pup liver. Fatty acid metabolism can be altered by desaturation or elongation reactions, which mainly occur in the liver [31]. We have earlier reported that maternal micronutrients like folic acid and vitamin B_12_ altered the levels of LCPUFA, activity and expression of fatty acid desaturases in the dam liver at end of pregnancy [23]. Thus, one of the possible reasons for the decrease in the DHA and ARA levels is the insufficient conversion of alpha linolenic acid (ALA) to DHA and linoleic acid (LA) to ARA, since we have observed increased ALA and LA in both the micronutrient deficient groups.

Omega-3 fatty acid supplementation to the micronutrient deficient diet normalized the expression of PPARγ in both BD and FD group while PPARα in BD group. These results are consistent with the earlier report which suggests that dietary LCPUFAs activate PPARα and PPARγ by increasing lipid oxidation [32]. Maternal DHA supplementation has also been reported to normalise intrauterine growth restriction (IUGR) induced visceral PPARγ expression in the male rats [33]. This interaction of PPARs with their ligands at the time of fetal development is important for the adaptation of long term lipid metabolism [34].

There was no change in the expression of pup liver SREBP-1c, LXRα and RXRα in the micronutrient deficient groups while omega-3 fatty acid supplementation decreased the expression of these transcription factors. This may be due to the fact that the omega-3 and omega 6-LCPUFAs are feed-forward activators of PPARs, while these same fatty acids are feedback inhibitors of LXRs and SREBPs [35]. These transcription factors are interdependent and the amount of these nuclear receptors in the nucleus and the concentrations of their ligands are the important factors in determining the overall effect [36]. Cell line studies have demonstrated that hepatic PPARα activation suppresses the LXR mediated expression of SREBP-1c and this inhibitory effect is enhanced by the addition of PPAR ligands. [10]. Similar effect was also seen due to decreased amount of LXR/RXR heterodimers and enhanced binding of PPARα to RXR [37]. Thus, the co-ordinated relationship between these transcription factors is critical for the regulation of downstream genes involved in the fatty acid metabolism.

In the current study, omega-3 fatty acid supplemented group had a lower birth weight. This may be because these omega-3 fatty acids are known to increase the lean mass of the body [38]–[40]. Further, omega-3 polyunsaturated fatty acids have also been reported to elicit a number of effects such as improvements in circulation which might facilitate nutrient delivery to skeletal muscles and changes in gene expression which shift metabolism towards increased accretion of lean tissue, enhanced fat oxidation and energy expenditure and reduced fat deposition [41].

A recent report indicates that changes in maternal dietary patterns influence the metabolic homeostasis in the liver and thereby play a central role in fetal programming [42]. In the current study, we observed higher global DNA methylation levels in the pup liver in both the micronutrient deficient groups. The levels of methylation were similar to those reported by us earlier where we have shown that altered micronutrients like folic acid and vitamin B_12_ during pregnancy cause global hypo DNA methylation in the placenta [21]. Folate and vitamin B_12_ are known to regulate the formation of S-adenosyl methionine (SAM) molecule which is the universal methyl donor for most of the reactions and any disturbance in the one carbon cycle affects cell proliferation and gene expression [43]. Reports indicate that altered methyl nutrition during peri-conception and early preimplantation development modifies the patterns of DNA methylation in adult tissues [44], [45]. Further, there are contradictory reports regarding the effect of methyl donor (methionine, folate, vitamin B_12_, choline) deficiency on the global DNA methylation status. Some reports suggest that these deficiencies lead to global DNA hypomethylation in various tissues like brain, liver, leukocyte etc [46]–[48] while others suggest that folic acid deficiency during pregnancy has no change in the global DNA methylation in the rat offspring [49].

A recent study have also shown that maternal omega-3 fatty acid availability during pregnancy and lactation alters the epigenetic status of fatty acid desaturases 2 (Fads2) in both mother and its offspring [50]. Thus altered expression of PPAR, SREBP-1c, LXRα and RXRα genes in the present study may possibly be due to low levels of pup liver DHA and altered promoter DNA methylation of these genes. Further studies are being carried out in our laboratory to examine gene specific methylation changes in these transcription factors. The current study has some limitations like the interaction between the gender and the diet has not been studied. In this study, the degree of fertility in various groups was not evaluated. Nevertheless, in our earlier study, adverse effects of micronutrient deficiencies resulted in abnormal estrous cycle which could be normalized by omega-3 fatty acid supplementation [27]. Also, there is a need to understand the effects of maternal micronutrient deficiency and omega-3 fatty acid supplementation on other transcription factors like hepatic nuclear factor-4α, nuclear factor kβ, glucocorticoid receptor involved in the hepatic fatty acid, cholesterol, carbohydrate and bile acid metabolism in the offspring. This will provide an insight into the role of one carbon cycle and its interaction with omega-3 fatty acids in the fetal lipid metabolism and the subsequent risk for the metabolic syndromes in the adults.

## Conclusion

Our data for the first time demonstrate that maternal folic acid or vitamin B_12_ deficiency from the pre-conception period alters the expression of hepatic transcription factors in the pup liver. Omega-3 fatty acid supplementation was found to be beneficial.
